# Affordable approach using flexor hallucis longus muscle graft for chronic Achilles tendon rupture: A case report

**DOI:** 10.1016/j.ijscr.2025.111470

**Published:** 2025-06-04

**Authors:** Romy Deviandri, Muhammad Wiranata, Richardo Winara, Fitri Denovinsa, Shalsabila Ghaasani, Nasywa Devina Mecca, Syarif Mas'ud

**Affiliations:** a Department of Orthopedics, University of Groningen, University Medical Center Groningen, Groningen, the Netherlands; b Faculty of Medicine, Universitas Riau, Arifin Achmad Hospital, Pekanbaru, Indonesia; c Department of Orthopedics, Universitas Padjadjaran, Hasan Sadikin Hospital, Bandung, Indonesia

**Keywords:** Tendon rupture, Cost-effective, Tendon graft, Surgery, Case report

## Abstract

**Introduction and importance:**

The Achilles tendon is vital in maintaining balance when walking. An affordable approach using the flexor hallucis longus (FHL) tendon graft is a cost-effective treatment.

**Case presentation:**

A 35-year-old male who worked as a porter was taken to the clinic after falling while going downstairs a year ago. The physical examination showed gastrocnemius muscle atrophy and limited range of motion (ROM). The Thompson test was performed, and the result was positive for the Achilles tendon rupture (ATR).

**Clinical discussion:**

Various techniques could be used in a chronic Achilles case, including autograft and allograft. Because the gap was 5.5 cm, we implemented a suitable surgical approach using an FHL tendon graft to repair a chronic ATR. The Euroqol 5-Dimension (EQ5D), the Visual Analog Scale (VAS), the Foot and Ankle Disability Index (FADI), and the cost-effectiveness (CEA) were measured to follow up patients' outcomes.

**Conclusion:**

A grafting procedure using the FHL tendon was affordable and cost-effective for reconstructing a chronic ATR. This technique was preferable, especially in remote area hospitals.

## Highlights

• A chronic Achilles tendon rupture should be repaired to improve patient's quality of life.
• FHL muscle grafting is cost-effective procedure.
• A grafting approach using FHL muscle is more applicable in remote area hospital.
• Foot and Ankle Disability Index (FADI) and Visual Analog Scale (VAS) can be used to evaluate the outcome.

## 1 Introduction

ATR is the most common tendon injury in individuals while engaging in activities requiring considerable leg movement, such as running and jumping [1]. This condition mostly affects male and elderly people [2]. ATR classified as acute and chronic, which is categorized as chronic when symptoms exist after 4–6 weeks [3]. Several options can be chosen, a direct end-to-end repair if the gap distance is acceptable, tendon advancement, V—Y alignment, turndown flaps, and tendon graft transfer [[4], [5], [6]]. Bharat et.al showed that V—Y tendon transfer with the ATR gaps ranging from 5 to 10 cm revealed an aesthetic appearance [5,6]. However, this approach is associated with a deficiency in peak torque and limited acceptability in the longer ATR gap. Adukia et al. implemented a quadriceps tendon graft on a cadaver model. This study describe that the biomechanical strength of the tendon is high, but it may affect the strength of the quadriceps [7]. The allograft approach could be an option in the longer ATR gap, but This approach needs a higher cost, and the availability of the graft is limited in several health centers.

This case reported a patient with chronic ATR treated using FHL tendon graft, which is cost-effective. Our report recorded on the SCARE 2025 guidelines [8].

## 2 Case presentation

A 35-year-old male working as a delivery courier was taken to the clinic with a chief complaint of mobilization difficulties. He felt discomfort around the right ankle area and inability to walk. A year ago, he had a history of falling to the ground while going downstairs. After that accident, the patient just took himself to the bonesetter. The patient was referred to the orthopedic unit because the complaint still existed and impaired his activities. There was no history of hospitalization, prolonged drug use, or any surgical intervention. The patient had been an active smoker for 10 years and had no history of allergy.

The patient came with a stable condition, hemodynamics within normal limits, and without signs of fever. Body Mass Index reveals overweight (BMI = 24,0 kg/m^2^). Physical examination reveals the gastrocnemius muscle atrophy on the posterior aspect of the knee. Palpation shows no tenderness. However, there was a gap in the posterior ankle area. The patient's ROM was restricted while performing plantar and dorsal flexion of the foot. A positive result was seen after performing the Thompson test examination. After thorough examination, the patient was diagnosed with a chronic ATR. Furthermore, a prompt surgical procedure must be performed to reach optimal gait function.

The intervention for this patient aimed to increase the ability for mobility and improve the patient's quality of life (QOL). After giving informed consent, the patient agreed to undergo operative surgery. During surgical preparation, the patient had no comorbid diseases such as Diabetic Mellitus and Hypertension. An orthopedic surgeon can implement a grafting procedure in our hospital. Spinal anesthesia was administered to the patient in a prone position. We also prepared for the instrument, including a non-absorbable suturing kit (Ethibond 2-0, Ethicon). He was provided Cefazolin 2 g/IV as a prophylactic wide-spectrum antibiotic.

**Fig. 1:**
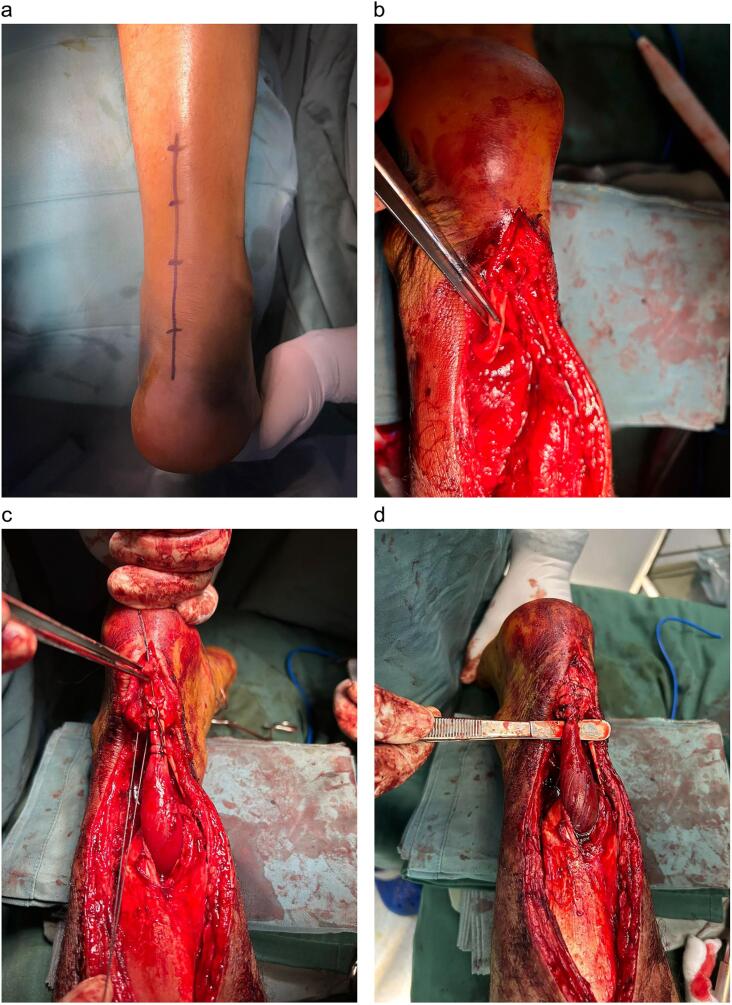
A marking procedure (a). Identification of FHL (b). The whipstitch suture was created at the edge of FHL and inserted inside the Achilles aponeurosis (c). FHL was attached to the calcaneus bone, and AT was completely attached (d).

First, the rupture was identified, and gap measurement was carried out. In this case, we found the gap was 5,5 cm from the insertion of AT. Based on this condition, the FHL tendon was chosen for grafting. After identifying FHL at the medial aspect of the ankle area, harvesting FHL was performed in the distal part as it entered the tarsal tunnel. Next, the whipstitch suture was created at the edge of the tendon with a non-absorbable suture (Ethibond 2–0, Ethicon) (Fig. 1). After inserting the FHL graft into the middle of the Achilles aponeurosis, each part was sutured by a simple suture. Finally, the distal part of the ATR draft was locked to the origin of the Achilles tendon in the calcaneus bone.

There were no intra- or postoperative complications. After surgery, an anterior splint was installed to immobilize the affected ankle. The patient was also advised to follow the physiotherapy program provided by the rehabilitation unit to improve sensory and motor function, avoid lifting weight-bearing objects, and maintain his BMI at a normal level.

The objectives of this Achilles treatment were to restore anatomic length and physiologic tension, supply sufficient strength for appropriate propulsion, maximize functional return to activity, reduce any related pain, and minimize any consequences. A proper post-operative program was conducted for the patient. In the first three weeks, swelling management with ice, compression, and elevation was promoted, while an early range of motion exercise was allowed to reduce the adhesion after three weeks.

Six weeks after surgery, the patient was advised to take down the anterior splint and start the range of motion exercise of the ankle area. The surgeon aims to achieve a balance of gait. The patient was trained to perform straight walking after three months. The patient felt improvement in his right ankle after performing several examinations post-surgery. There are no specific complaints while performing plantar and dorsal flexion or walking. Mild pain and tenderness were still found, but there was an improvement in the VAS score, from 8 to 1 after surgery and three months after surgery, respectively. Four months after surgery, the VAS score shows zero while performing specific performances such as walking downstairs, zig-zag walking, and running.

An FADI score was also used to evaluate the patient's outcome. The patient scored 50 before surgery and increased to 99 six months after surgery. The EQ5D scores before and the six-month follow-up after surgery were 0.5 and 0.9, respectively. Both the FADI and EQ5D scores showed promising results.

## 3 Discussion

Achilles Tendon (AT) is a common rupture on the lower extremity. The incidence rate is approximately 7 to 40 cases per 100,000 person-years. This statistic made ATR a prevalent injury in orthopedic cases [9]. Multiple factors contribute to Achilles tendon rupture, including biomechanical, histological, and genetic influences. [10]

Recurrent rupture of AT shows a higher risk of having late complications, with about 5 % of patients suffering tendinitis after open surgery by the surgeon. A study shows 5,6 % rupture of AT associated with deep infection. Our report shows no specific complaints about the latest surgery due to a rupture of AT. In every rehabilitation phase, the patient feels slight pain without a fever. On the last day of the rehabilitation examination, the patient's visual analog scale remained stable, scoring 0–1.^8.^

A prior Magnetic Resonance Imaging (MRI) or Ultrasound is essential for identifying and diagnosing of ATR. Besides, it is useful for measuring the gap of ATR and determining the proper treatment. The ultrasound could be used as an alternative imaging option rather than MRI in the condition of limited resources. In our case, there was no radiology examination using MRI due to the limited facilities in our hospital, so the ultrasound was used and provided useful information for determining the further treatment [11,12].

Surgical intervention of ATR can be performed using either a percutaneous technique or open repair. Both methods have their advantages and disadvantages. Percutaneous repair is a minimally invasive procedure, but it increases the risk of severe nerve injury and rupture. Besides, it is more useful in acute conditions where the gap ATR is minimal. Furthermore, due to the lack of visualization, this technique has a higher re-rupture rate, while open surgery reduces the risk of re-rupture [13].

In the initial step of surgery, after administering anesthesia and the patient is positioned prone, and a 6–10 cm incision is made medially along the AT to avoid sural nerve injury. Primary repair or grafting can be performed, depending on the tendon gap. Myerson classification correlate the gap and corresponding treatment recommendations, where the type 1 with the gap less than 2 cm could be treated with end to end repair, then type 2 with the gap within 2-5 cm could be treated with V—Y lengthening with or without tendon transfer, and the type 3 with the gap more than 5 cm could be treated with tendon transfer, either by autograft or allograft [14].

In this case, a tendon graft using the FHL muscle was made due to a gap found to be 5.5 cm. Feng et al. suggested that when the tendon gap reaches 3–6 cm, a local tendon transfer may be required, either using the peroneus brevis tendon or FHL. Compared to the peroneus brevis tendon graft, the FHL muscle graft shows better results, although the difference is statistically insignificant. Patients who undergo a peroneus brevis tendon graft can still participate in sports and daily activities, but report decreased plantar flexion strength. Patients who undergo an FHL muscle graft approach near-normal maximum strength but experience reduced endurance [5]. Besides using the autograft, an allograft could be used instead. However, this technique needs specific preparation and limited availability in some health centers, including our local hospital. Furthermore, this technique is relatively expensive, with an additional cost of using the Achilles allograft of USD 615 [15].

A case series conducted by Abubeih et al. on 21 patients suffering from chronic ATR, FHL muscle graft may improve patients' quality of life, including results of the American Orthopaedic Foot and Ankle Society Ankle-Hindfoot Scale (AOFAS-AH) [16]. They can continue daily activities without specific limitations. A study by Ahmad et al. showed that in 32 patients who had done FHL grafting, all patients could have done a single heel raise without any weakness in a mean of 62.3 months of follow-up [17].

Open repair carries a higher risk of wound complications, such as wound dehiscence and infection, so preventing and managing infection is essential. In this case, cefazolin 2 g as a broad-spectrum prophylaxis antibiotic was administered as a guideline in our local hospital.

A conservative modality with cast immobilization could be used in chronic ATR. A conservative treatment could reduce the risk of infection that threatens in a case of open surgery. This treatment aims to improve function without surgery. However, a conservative treatment was chosen only in chronic partial ATR, or in the acute condition when the gap is no longer than 2 cm. This was not the case in this study. An advanced surgical approach with the allograft or autograft was recommended if the gap was over 5 cm. Regarding the locked method in the calcaneus, either biotenodesis screw or suture anchor shows promising and favorable results, especially on the athletes, but this procedure is more expensive and categorized as depending on instrument availabilities [18].

Based on our report, a grafting procedure using an FHL tendon attached to the calcaneus bone by direct suture to the bone by needle (Ethibond 2–0, Ethicon) was more suitable and affordable for this patient in a hospital setting. The hospital costs incurred per procedure in our local's hospital —consist of bed cost, pharmacy, and surgery cost— was USD 600, resulting in a cost-effective FHL tendon graft in ATR cases of 1500/QALY.

Other modalities can also be performed, such as using allografts that are popular in some regions. However, besides this preparation being specific and needing more resources, the allograft's prices are also higher among other techniques, whereas using the Achilles tendon allograft and bone-patellar tendon graft requires an additional direct cost of USD 615 and USD 800, respectively, as the sum price of the graft [15]. So, due to its cost-effectiveness, the FHL graft procedure is preferable in peripheral areas, depending on financial status and the limited budget for particular cases [13].

External support, such as taping and bracing, is used for chronic ankle instability, including ATR. The research found that these supports reduce the risk of ankle instability. External support provides biomechanical support by reducing plantar flexion angle [19,20]. This guarantees the ankle becomes stable and promotes the injury's healing, resulting in a good outcome.

Evaluation was needed to improve the outcome of ATR after surgery. Choi et al. used the FADI score to evaluate ankle stability during recovery. FADI was a good instrument for assessing ankle instability, including chronic ATR. FADI was used to detect if there was still disability in patients with ATR. This instrument was sensitive to improve the function of the patient's ankle during rehabilitation [21]. On this patient, we scored 50 before surgery and 99 post-surgery. This score shows improvement in patients between pre-surgery and post-surgery.

## 4 Conclusion

In a case of Chronic ATR, a suitable grafting procedure by FHL muscle showed a good functional outcome. This technique was cost-effective and very applicable in remote area hospitals.

## Consent

The patient agreed to publish this report after obtaining informed consent. A copy of the consent form is available for review by the Editor-in-Chief upon request.

## Ethical approval

This case report does not require ethical approval.

## Guarantor

Romy Deviandri

## Research registration number

In this study, the reported case was not a “First in man” studies.

## Funding

This report did not reveal any specific funding from commercial or public sources.

## Author contribution

The contribution made by this report of all authors.

## Conflict of interest statement

All authors have no conflicts of interest.
